# Correction: Re-examining the effects of drought on intimate-partner violence

**DOI:** 10.1371/journal.pone.0307314

**Published:** 2024-07-11

**Authors:** Matthew Cooper, Austin Sandler, Sveva Vitellozzi, Yeyoung Lee, Greg Seymour, Beliyou Haile, Carlo Azzarri

The seventh author’s name is spelled incorrectly. The correct name is: Carlo Azzarri.

The following information is missing from the Funding statement: This research was financially supported by the Gender, Climate Change, and Nutrition Integration (GCAN) project, an initiative supported by the United States Agency for International Development (USAID), implemented under the CGIAR Research Program on Climate Change, Agriculture and Food Security (CCAFS), and led by Claudia Ringler.
