# Supplementary material for: Re-examining the effects of drought on intimate-partner violence
Source: PLoS One. 2021 Jul 20;16(7):e0254346. doi: 10.1371/journal.pone.0254346 (PMC8291644; doi:10.1371/journal.pone.0254346)
Supplement: S1 File — (PDF) [file pone.0254346.s001.pdf]

S1 File

Summary statistics

S1 Table. Count of Observations by Country and Year (Pt. 1).

	2000	2003	2004	2005	2006	2007	2008	2009	2010
Angola	0	0	0	0	0	0	0	0	0
Benin	0	0	0	0	0	0	0	0	0
Burkina Faso	0	0	0	0	0	0	0	0	9028
Burundi	0	0	0	0	0	0	0	0	0
Cambodia	2077	0	0	1304	705	0	0	0	0
Cameroon	0	0	2223	0	0	0	0	0	0
Chad	0	0	0	0	0	0	0	0	0
Comoros	0	0	0	0	0	0	0	0	0
Congo - Kinshasa	0	0	0	0	0	0	0	0	0
Côte d'Ivoire	0	0	0	0	0	0	0	0	0
Dominican Republic	0	0	0	0	0	6241	0	0	0
Ethiopia	0	0	0	0	0	0	3898	0	0
Gabon	0	0	0	0	0	0	0	0	0
Ghana	0	0	0	0	0	0	1469	0	0
Guatemala	0	0	0	0	0	0	0	0	0
Haiti	2085	0	0	857	1323	0	0	0	0
Honduras	0	0	0	0	0	0	0	0	0
India	0	0	0	0	0	0	0	0	0
Kenya	0	0	0	0	0	0	1915	2294	0
Liberia	0	0	0	0	261	3105	0	0	0
Malawi	0	0	5522	1822	0	0	0	0	4410
Mali	0	0	0	0	8515	0	0	0	0
Mozambique	0	0	0	0	0	0	0	0	0
Myanmar (Burma)	0	0	0	0	0	0	0	0	0
Namibia	0	0	0	0	0	0	0	0	0
Nepal	0	0	0	0	0	0	0	0	0
Nigeria	0	0	0	0	0	0	17555	0	0
Pakistan	0	0	0	0	0	0	0	0	0
Peru	0	176	2690	2913	3259	3159	3008	12038	0
Philippines	0	0	0	0	0	0	6693	0	0
Rwanda	0	0	0	2275	0	0	0	0	0
Sierra Leone	0	0	0	0	0	0	0	0	0
South Africa	0	0	0	0	0	0	0	0	0
Tajikistan	0	0	0	0	0	0	0	0	0
Tanzania	0	0	0	0	0	0	0	429	4317
Timor-Leste	0	0	0	0	0	0	0	1684	364
Togo	0	0	0	0	0	0	0	0	0
Uganda	0	0	0	0	1318	0	0	0	0
Zambia	0	0	0	0	0	3543	0	0	0
Zimbabwe	0	0	0	2936	998	0	0	0	2250
Sum	4162	176	10435	12107	16379	16048	34538	16445	20369

S2 Table. Count of Observations by Country and Year (Pt. 2)

	2011	2012	2013	2014	2015	2016	2017	2018	Sum
Angola	0	0	0	0	3010	3000	0	0	6010
Benin	0	0	0	0	0	0	1951	1926	3877
Burkina Faso	0	0	0	0	0	0	0	0	9028
Burundi	0	0	0	0	0	3752	2600	0	6352
Cambodia	0	0	0	3200	0	0	0	0	7286
Cameroon	3428	0	0	0	0	0	0	0	5651
Chad	0	0	0	989	2242	0	0	0	3231
Comoros	0	2085	0	0	0	0	0	0	2085
Congo - Kinshasa	0	0	3481	1075	0	0	0	0	4556
Côte d'Ivoire	406	3733	0	0	0	0	0	0	4139
Dominican Republic	0	0	5001	0	0	0	0	0	11242
Ethiopia	0	0	0	0	0	0	0	0	3898
Gabon	0	3117	0	0	0	0	0	0	3117
Ghana	0	0	0	0	0	0	0	0	1469
Guatemala	0	0	0	1860	3811	0	0	0	5671
Haiti	0	5565	0	0	0	712	3050	0	13592
Honduras	4488	5725	0	0	0	0	0	0	10213
India	0	0	0	0	30500	31632	0	0	62132
Kenya	0	0	0	3741	0	0	0	0	7950
Liberia	0	0	0	0	0	0	0	0	3366
Malawi	0	0	0	0	3076	1457	0	0	16287
Mali	0	1850	1188	0	0	0	0	0	11553
Mozambique	4592	0	0	0	0	0	0	0	4592
Myanmar (Burma)	0	0	0	0	546	2521	0	0	3067
Namibia	0	0	1191	0	0	0	0	0	1191
Nepal	3335	0	0	0	0	3601	62	0	6998
Nigeria	0	0	20479	0	0	0	0	0	38034
Pakistan	0	0	0	0	0	0	1103	2852	3955
Peru	0	0	0	0	0	0	0	0	27243
Philippines	0	0	0	0	0	0	0	0	6693
Rwanda	0	0	0	596	1020	0	0	0	3891
Sierra Leone	0	0	3968	0	0	0	0	0	3968
South Africa	0	0	0	0	0	1899	0	0	1899
Tajikistan	0	0	0	0	0	0	4897	0	4897
Tanzania	0	0	0	0	4842	1597	0	0	11185
Timor-Leste	0	0	0	0	0	3571	0	0	5619
Togo	0	0	1803	2929	0	0	0	0	4732
Uganda	1391	0	0	0	0	6101	0	0	8810
Zambia	0	0	4480	3089	0	0	0	0	11112
Zimbabwe	1875	0	0	0	4778	0	0	0	12837
Sum	19515	22075	41591	17479	53825	59843	13663	4778	363428

Moran's I test results

The following tables show the Moran's I test statistic of spatial autocorrelation for models across all three continents and all four types of IPV. The expected value of the Moran's I test under the null hypothesis of no spatial autocorrelation tends towards 0 as data size increases. Thus, as the models incorporate increasingly sophisticated spatial splines, the test statistic is indicative of no spatial autocorrelation as it gets closer to 0. Models were fit with increasingly complex spatial splines until there was no longer significant autocorrelation in the residuals at $\alpha = 0.05$. (*$p < 0.05$, **$p < 0.01$, ***$p < 0.001$).

S3 Table. Moran's I test statistic for regressions for controlling behaviors.

	Controlling Behaviors		
	SSA	*Asia*	*LAC*
No Spatial Terms	0.0054 ***	0.0187 ***	0.0028 ***
50-knot Thin Plate Splines on a Sphere	0.0038 ***	0.0065 ***	0.0001
100-knot Thin Plate Splines on a Sphere	0.003 ***	0.0045 ***	
500-knot Thin Plate Splines on a Sphere	0.0006 ***	0.0022 ***	
1000-knot Thin Plate Splines on a Sphere	-0.0001	0.0009 ***	
1500-knot Thin Plate Splines on a Sphere		0.0002	

S4 Table. Moran's I test statistic for regressions for emotional violence.

	Emotional Violence		
	SSA	*Asia*	*LAC*
No Spatial Terms	0.0066 ***	0.0056 ***	0.0021 ***
50-knot Thin Plate Splines on a Sphere	0.003 ***	0.0024 ***	0.0004
100-knot Thin Plate Splines on a Sphere	0.0022 ***	0.0015 ***	
500-knot Thin Plate Splines on a Sphere	0.0007 ***	0.0005 ***	
1000-knot Thin Plate Splines on a Sphere	0.0001	0.0003	
1500-knot Thin Plate Splines on a Sphere			

S5 Table. Moran's I test statistic for regressions for physical violence.

	Physical Violence		
	SSA	*Asia*	*LAC*
No Spatial Terms	0.0069 ***	0.009 ***	0.0032 ***
50-knot Thin Plate Splines on a Sphere	0.0026 ***	0.0028 ***	0.0009 *
100-knot Thin Plate Splines on a Sphere	0.0016 ***	0.0017 ***	0.0005
500-knot Thin Plate Splines on a Sphere	0.0004 *	0.0002	
1000-knot Thin Plate Splines on a Sphere	0.0001		
1500-knot Thin Plate Splines on a Sphere			

S6 Table. Moran's I test statistic for regressions for sexual violence.

	Sexual Violence		
	SSA	*Asia*	*LAC*
No Spatial Terms	0.0037 ***	0.0024 ***	0.0013 ***
50-knot Thin Plate Splines on a Sphere	0.0009 ***	0.0014 ***	0.0004
100-knot Thin Plate Splines on a Sphere	0.0007 ***	0.0008 ***	
500-knot Thin Plate Splines on a Sphere	0	0.0003	
1000-knot Thin Plate Splines on a Sphere			
1500-knot Thin Plate Splines on a Sphere			

Comparisons with models with no spatial terms

Full model results

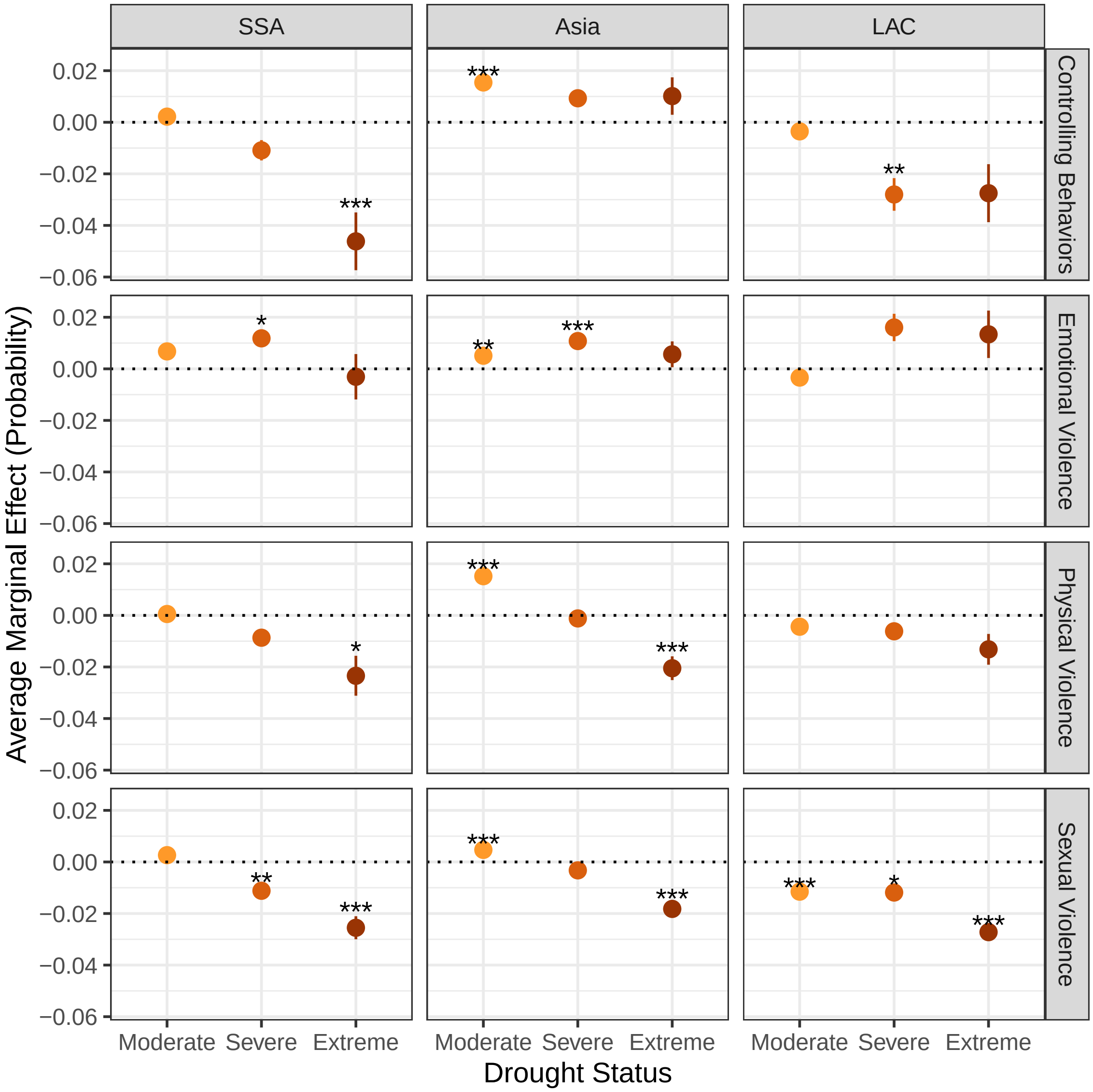

S1 Fig. Results for models with no spatial terms. Average marginal effect of a moderate, severe, or extreme drought on a woman's probability of experiencing IPV relative to non-drought conditions, for all four types of IPV across three different sized data sets. (Significance values are Bonferroni-corrected for testing 12 hypotheses, so $*p < 0.05/12$, $**p < 0.01/12$, $***p < 0.001/12$.)

S7 Table. Controlling Behaviors AMEs. Average marginal effects of covariates on controlling behaviors in models with (*sp.*) and without (*asp.*) spatial terms across all three continents. (*$p < 0.05$, **$p < 0.01$, ***$p < 0.001$)

	SSA		Asia		LAC	
	asp.	*sp.*	*asp.*	*sp.*	*asp.*	*sp.*
Drought (Moderate)	-0.001	-0.009**	0.036***	0.024***	0.017**	0.009
	(0.003)	(0.004)	(0.004)	(0.006)	(0.006)	(0.007)
Drought (Severe)	0.023***	-0.004	0.014	0.01	0.032***	0.041***
	(0.005)	(0.007)	(0.009)	(0.013)	(0.008)	(0.009)
Drought (Extreme)	0.005	-0.048***	0.023	-0.026	0.042***	0.052***
	(0.009)	(0.013)	(0.017)	(0.025)	(0.011)	(0.012)
Woman is Married	-0.058***	-0.057***	-0.087***	-0.085***	-0.077***	-0.083***
	(0.004)	(0.004)	(0.012)	(0.012)	(0.004)	(0.004)
Woman is Literate	0.013***	0.021***	-0.054***	-0.021***	0.015**	0.02***
	(0.003)	(0.003)	(0.004)	(0.004)	(0.005)	(0.005)
Woman's Age (20 - 29)	-0.004	0.006	-0.014	0.001	-0.043***	-0.044***
	(0.005)	(0.005)	(0.01)	(0.01)	(0.009)	(0.009)
Woman's Age (30 - 39)	-0.032***	-0.015**	-0.032**	-0.008	-0.067***	-0.068***
	(0.006)	(0.006)	(0.011)	(0.011)	(0.01)	(0.01)
Woman's Age (40 - 49)	-0.075***	-0.056***	-0.07***	-0.046***	-0.104***	-0.106***
	(0.007)	(0.007)	(0.012)	(0.011)	(0.012)	(0.012)
Number of Births (0 - 2)	0.007	0.006	-0.017**	-0.016**	-0.003	-0.006
	(0.005)	(0.005)	(0.006)	(0.006)	(0.008)	(0.008)
Number of Births (3 - 4)	0.009.	0.006	0.027***	0.003	0.027**	0.022*
	(0.005)	(0.005)	(0.007)	(0.007)	(0.009)	(0.009)
Number of Births (> 4)	0.016**	0.01.	0.059***	0.022**	0.064***	0.055***
	(0.006)	(0.005)	(0.008)	(0.008)	(0.009)	(0.009)
Partner Education (None)	0.01*	0.005	0.058***	0.062***	0.013	0.022*
	(0.005)	(0.005)	(0.006)	(0.006)	(0.01)	(0.01)
Partner Education (Primary)	0.027***	0.026***	0.036***	0.057***	0.046***	0.052***
	(0.005)	(0.005)	(0.006)	(0.006)	(0.007)	(0.007)
Partner Education (Secondar)	0.03***	0.029***	0.026***	0.043***	0.044***	0.045***
	(0.004)	(0.004)	(0.005)	(0.005)	(0.006)	(0.006)
Partner Age (20 - 29)	0.047**	0.052**	0.001	0.009	-0.05*	-0.048*
	(0.016)	(0.016)	(0.023)	(0.022)	(0.021)	(0.021)
Partner Age (30 - 39)	0.04*	0.047**	-0.038.	-0.01	-0.064**	-0.061**
	(0.016)	(0.016)	(0.023)	(0.022)	(0.022)	(0.022)
Partner Age (40 - 49)	0.04*	0.046**	-0.053*	-0.014	-0.071***	-0.065**
	(0.017)	(0.016)	(0.023)	(0.022)	(0.022)	(0.022)
Partner Age (> 49)	0.024	0.031.	-0.072**	-0.017	-0.075***	-0.067**
	(0.017)	(0.017)	(0.023)	(0.023)	(0.023)	(0.023)
Household Size (4 - 5)	-0.002	-0.003	-0.008.	-0.01*	-0.006	-0.005
	(0.003)	(0.003)	(0.004)	(0.004)	(0.005)	(0.005)
Household Size (> 5)	0.004	-0.001	0.006	-0.008	-0.014*	-0.011*
	(0.003)	(0.003)	(0.005)	(0.005)	(0.006)	(0.006)
Household is Rural	-0.025***	-0.031***	0	0.002	-0.014***	-0.022***
	(0.003)	(0.003)	(0.003)	(0.004)	(0.004)	(0.004)
AIC	238905.174	231344.118	131757.018	123790.054	84996.492	84586.621

S8 Table. Sexual Violence AMEs. Average marginal effects of covariates on sexual violence in models with (*sp.*) and without (*asp.*) spatial terms across all three continents. (*$p < 0.05$, **$p < 0.01$, ***$p < 0.001$)

	SSA		Asia		LAC	
	asp.	*sp.*	*asp.*	*sp.*	*asp.*	*sp.*
Drought (Moderate)	-0.002	0.001	0.001	-0.003	-0.008**	-0.007*
	(0.002)	(0.002)	(0.002)	(0.002)	(0.003)	(0.003)
Drought (Severe)	-0.003	-0.003	0.009*	-0.004	-0.006	-0.001
	(0.003)	(0.004)	(0.005)	(0.005)	(0.005)	(0.005)
Drought (Extreme)	-0.014*	-0.013.	0.015*	-0.003	-0.025***	-0.02**
	(0.006)	(0.007)	(0.009)	(0.009)	(0.005)	(0.006)
Woman is Married	-0.016***	-0.014***	-0.036***	-0.035***	-0.009***	-0.01***
	(0.002)	(0.002)	(0.005)	(0.005)	(0.002)	(0.002)
Woman is Literate	0.006***	0.003.	-0.011***	-0.008***	0.002	0.003
	(0.002)	(0.002)	(0.002)	(0.002)	(0.002)	(0.002)
Woman's Age (20 - 29)	-0.006.	-0.006.	-0.005	-0.001	-0.016**	-0.016**
	(0.003)	(0.003)	(0.005)	(0.005)	(0.005)	(0.005)
Woman's Age (30 - 39)	-0.013***	-0.01**	-0.01*	-0.005	-0.023***	-0.023***
	(0.003)	(0.003)	(0.005)	(0.005)	(0.004)	(0.004)
Woman's Age (40 - 49)	-0.024***	-0.022***	-0.018***	-0.011*	-0.029***	-0.029***
	(0.004)	(0.004)	(0.005)	(0.005)	(0.004)	(0.004)
Number of Births (0 - 2)	0.015***	0.014***	0.004	0.004	0.009**	0.009**
	(0.003)	(0.003)	(0.003)	(0.003)	(0.003)	(0.003)
Number of Births (3 - 4)	0.025***	0.023***	0.015***	0.013***	0.021***	0.021***
	(0.004)	(0.004)	(0.004)	(0.004)	(0.005)	(0.005)
Number of Births (> 4)	0.033***	0.029***	0.02***	0.015***	0.039***	0.038***
	(0.004)	(0.004)	(0.005)	(0.004)	(0.007)	(0.007)
Partner Education (None)	0.019***	0.026***	0.037***	0.036***	0.015***	0.015***
	(0.003)	(0.004)	(0.004)	(0.004)	(0.005)	(0.005)
Partner Education (Primary)	0.038***	0.036***	0.031***	0.031***	0.025***	0.025***
	(0.004)	(0.004)	(0.004)	(0.004)	(0.004)	(0.005)
Partner Education (Secondar)	0.025***	0.023***	0.018***	0.019***	0.017***	0.017***
	(0.003)	(0.003)	(0.003)	(0.003)	(0.004)	(0.004)
Partner Age (20 - 29)	-0.002	-0.006	-0.004	-0.002	0.002	0.002
	(0.01)	(0.01)	(0.011)	(0.01)	(0.011)	(0.011)
Partner Age (30 - 39)	-0.01	-0.012	-0.004	-0.001	0.003	0.002
	(0.01)	(0.01)	(0.011)	(0.011)	(0.011)	(0.011)
Partner Age (40 - 49)	-0.016.	-0.018.	-0.009	-0.006	-0.001	-0.001
	(0.009)	(0.009)	(0.01)	(0.01)	(0.011)	(0.011)
Partner Age (> 49)	-0.02*	-0.023*	-0.015	-0.011	-0.008	-0.008
	(0.009)	(0.009)	(0.009)	(0.009)	(0.009)	(0.009)
Household Size (4 - 5)	0.003	0.001	-0.003	-0.003	0.002	0.002
	(0.002)	(0.002)	(0.002)	(0.002)	(0.002)	(0.002)
Household Size (> 5)	0.007***	0.003.	-0.003	-0.003	-0.001	-0.001
	(0.002)	(0.002)	(0.002)	(0.002)	(0.003)	(0.003)
Household is Rural	0.007***	0	0.001	-0.001	-0.007***	-0.009***
	(0.002)	(0.002)	(0.002)	(0.002)	(0.002)	(0.002)
AIC	109117.637	105537.595	38041.221	37094.177	25343.911	25247.616

S9 Table. Emotional Violence AMEs. Average marginal effects of covariates on emotional violence in models with (*sp.*) and without (*asp.*) spatial terms across all three continents. (*$p < 0.05$, **$p < 0.01$, ***$p < 0.001$)

	SSA		Asia		LAC	
	asp.	*sp.*	*asp.*	*sp.*	*asp.*	*sp.*
Drought (Moderate)	0	0.004	0.007*	0.009*	-0.009.	-0.004
	(0.003)	(0.003)	(0.003)	(0.004)	(0.005)	(0.005)
Drought (Severe)	0.001	0	-0.003	-0.002	0.001	0.015*
	(0.005)	(0.006)	(0.006)	(0.009)	(0.007)	(0.008)
Drought (Extreme)	-0.02*	-0.021*	0.018	0.02	-0.012	0.008
	(0.008)	(0.01)	(0.013)	(0.017)	(0.01)	(0.011)
Woman is Married	-0.036***	-0.031***	-0.057***	-0.054***	-0.045***	-0.045***
	(0.003)	(0.003)	(0.007)	(0.007)	(0.003)	(0.003)
Woman is Literate	0.002	-0.003	-0.024***	-0.023***	0.015***	0.017***
	(0.002)	(0.002)	(0.002)	(0.002)	(0.004)	(0.004)
Woman's Age (20 - 29)	0.016***	0.016***	-0.017*	-0.013.	-0.007	-0.008
	(0.005)	(0.004)	(0.007)	(0.007)	(0.008)	(0.008)
Woman's Age (30 - 39)	0.001	0.004	-0.024**	-0.017*	-0.03***	-0.029***
	(0.005)	(0.005)	(0.008)	(0.008)	(0.008)	(0.008)
Woman's Age (40 - 49)	-0.013*	-0.008	-0.042***	-0.033***	-0.041***	-0.04***
	(0.005)	(0.005)	(0.007)	(0.007)	(0.009)	(0.009)
Number of Births (0 - 2)	0.044***	0.045***	0.028***	0.026***	0.034***	0.035***
	(0.004)	(0.004)	(0.004)	(0.004)	(0.007)	(0.007)
Number of Births (3 - 4)	0.069***	0.067***	0.044***	0.043***	0.065***	0.064***
	(0.005)	(0.005)	(0.005)	(0.005)	(0.008)	(0.008)
Number of Births (> 4)	0.087***	0.082***	0.058***	0.056***	0.104***	0.1***
	(0.006)	(0.006)	(0.007)	(0.007)	(0.01)	(0.01)
Partner Education (None)	0.02***	0.036***	0.062***	0.059***	0.036***	0.043***
	(0.004)	(0.005)	(0.005)	(0.005)	(0.008)	(0.009)
Partner Education (Primary)	0.053***	0.049***	0.052***	0.051***	0.045***	0.047***
	(0.005)	(0.004)	(0.005)	(0.005)	(0.006)	(0.006)
Partner Education (Secondar)	0.034***	0.033***	0.028***	0.031***	0.034***	0.034***
	(0.004)	(0.004)	(0.003)	(0.003)	(0.006)	(0.005)
Partner Age (20 - 29)	0.009	0.009	0.007	0.008	0.018	0.018
	(0.015)	(0.015)	(0.016)	(0.016)	(0.018)	(0.018)
Partner Age (30 - 39)	0.004	0.008	0.008	0.007	0.017	0.016
	(0.015)	(0.015)	(0.016)	(0.016)	(0.018)	(0.018)
Partner Age (40 - 49)	0.003	0.008	0.009	0.005	0.01	0.009
	(0.015)	(0.015)	(0.017)	(0.016)	(0.018)	(0.018)
Partner Age (> 49)	-0.001	0.001	0.006	0.002	0.006	0.006
	(0.015)	(0.015)	(0.017)	(0.016)	(0.018)	(0.018)
Household Size (4 - 5)	0.007**	0.005.	-0.013***	-0.012***	0.008*	0.01*
	(0.003)	(0.003)	(0.003)	(0.003)	(0.004)	(0.004)
Household Size (> 5)	0.009**	0.002	-0.017***	-0.016***	-0.008.	-0.004
	(0.003)	(0.003)	(0.003)	(0.003)	(0.004)	(0.004)
Household is Rural	0.001	-0.004.	-0.004.	-0.003	-0.041***	-0.046***
	(0.002)	(0.002)	(0.002)	(0.002)	(0.003)	(0.003)
AIC	191435.38	184754.71	70331.545	68171.712	63928.606	63642.791

S10 Table. Physical Violence AMEs. Average marginal effects of covariates on physical violence in models with (*sp.*) and without (*asp.*) spatial terms across all three continents. (*$p < 0.05$, **$p < 0.01$, ***$p < 0.001$)

	SSA		Asia		LAC	
	asp.	*sp.*	*asp.*	*sp.*	*asp.*	*sp.*
Drought (Moderate)	-0.002	0.002	0.019***	0.002	-0.006	-0.006
	(0.002)	(0.003)	(0.003)	(0.004)	(0.004)	(0.004)
Drought (Severe)	0.011*	0.011*	0.007	-0.01	0.001	0.011.
	(0.005)	(0.006)	(0.007)	(0.009)	(0.006)	(0.007)
Drought (Extreme)	0.002	0.003	0.025.	-0.018	-0.019*	-0.002
	(0.008)	(0.01)	(0.014)	(0.015)	(0.008)	(0.01)
Woman is Married	-0.044***	-0.036***	-0.062***	-0.056***	-0.048***	-0.048***
	(0.003)	(0.003)	(0.011)	(0.01)	(0.002)	(0.003)
Woman is Literate	-0.006**	-0.013***	-0.057***	-0.047***	-0.005	-0.003
	(0.002)	(0.002)	(0.003)	(0.003)	(0.003)	(0.003)
Woman's Age (20 - 29)	0.019***	0.013**	-0.001	0.009	-0.029***	-0.03***
	(0.004)	(0.004)	(0.009)	(0.009)	(0.006)	(0.006)
Woman's Age (30 - 39)	0.001	-0.005	-0.015	0.003	-0.051***	-0.051***
	(0.005)	(0.005)	(0.009)	(0.009)	(0.006)	(0.006)
Woman's Age (40 - 49)	-0.013*	-0.018***	-0.038***	-0.018.	-0.071***	-0.071***
	(0.005)	(0.005)	(0.009)	(0.009)	(0.006)	(0.006)
Number of Births (0 - 2)	0.047***	0.046***	0.047***	0.047***	0.019***	0.019***
	(0.004)	(0.004)	(0.005)	(0.005)	(0.005)	(0.005)
Number of Births (3 - 4)	0.072***	0.069***	0.078***	0.075***	0.046***	0.045***
	(0.005)	(0.005)	(0.006)	(0.006)	(0.007)	(0.007)
Number of Births (> 4)	0.089***	0.084***	0.103***	0.097***	0.082***	0.078***
	(0.006)	(0.005)	(0.008)	(0.008)	(0.009)	(0.009)
Partner Education (None)	0.035***	0.062***	0.108***	0.103***	0.025***	0.032***
	(0.004)	(0.005)	(0.006)	(0.006)	(0.007)	(0.007)
Partner Education (Primary)	0.076***	0.074***	0.096***	0.099***	0.032***	0.036***
	(0.005)	(0.005)	(0.006)	(0.006)	(0.005)	(0.005)
Partner Education (Secondar)	0.059***	0.056***	0.052***	0.058***	0.026***	0.026***
	(0.004)	(0.004)	(0.004)	(0.004)	(0.005)	(0.005)
Partner Age (20 - 29)	-0.01	-0.005	0.018	0.016	-0.017	-0.017
	(0.015)	(0.015)	(0.022)	(0.021)	(0.014)	(0.014)
Partner Age (30 - 39)	-0.037*	-0.027.	0.007	0.005	-0.036*	-0.036**
	(0.014)	(0.014)	(0.021)	(0.021)	(0.013)	(0.013)
Partner Age (40 - 49)	-0.057***	-0.045**	-0.009	-0.013	-0.05***	-0.05***
	(0.013)	(0.013)	(0.02)	(0.02)	(0.012)	(0.012)
Partner Age (> 49)	-0.073***	-0.064***	-0.029	-0.03	-0.067***	-0.066***
	(0.013)	(0.012)	(0.019)	(0.019)	(0.011)	(0.011)
Household Size (4 - 5)	0.007**	0.005.	-0.013***	-0.011**	-0.005	-0.004
	(0.003)	(0.003)	(0.004)	(0.004)	(0.003)	(0.003)
Household Size (> 5)	0.006*	0.002	-0.022***	-0.022***	-0.02***	-0.018***
	(0.003)	(0.003)	(0.004)	(0.004)	(0.004)	(0.004)
Household is Rural	-0.002	-0.008***	-0.001	0.004	-0.031***	-0.034***
	(0.002)	(0.002)	(0.003)	(0.003)	(0.003)	(0.003)
AIC	182009.771	175307.867	92744.756	88650.629	49017.348	48698.885
