# Supplementary material for: Re-examining the effects of drought on intimate-partner violence
Source: PLoS One. 2021 Jul 20;16(7):e0254346. doi: 10.1371/journal.pone.0254346 (PMC8291644; doi:10.1371/journal.pone.0254346)
Supplement: Attachment — Submitted filename: response_to_reviewers.pdf [file pone.0254346.s002.pdf]

Response to Reviewers

Reviewer Notes:

Reviewer #1:

Alex McConnachie, Statistical Review

The paper by Cooper et al resolves the differing results between two previous papers looking at the association between periods of drought and intimate partner violence. They show that accounting for spatial autocorrelation is key, and that the associations between drought and IPV are far fewer than they appear when this is not accounted for.

The authors explain the methods and present the results from the different models very clearly.

No response.

Reviewer #2:

This paper studies the impact of precipitation variation on intimate partner violence, using DHS data from across the developing world. The question is important as IPV is common in much of the world and its overall drivers still poorly understood. As the authors point out, current evidence on links between climate and IPV — while plausible given broader links between climate and various types of conflict — is contradictory. The authors build a larger sample than past work and more carefully control for spatial autocorrelation, which is potentially important since (as they point out), both drought and IPV are likely spatially covariate. It's a nice paper and an important new piece of evidence on this question. I hope my questions/suggestions help improve the manuscript.

1. Does 12-month precipitation anomaly really capture the main mechanisms of interest here? A primary story has to do with rainfall-induced changes in agricultural productivity affecting female incomes and thus position within the household , but not all women are farmers, crops are not grown in all months, and temperature is as (or more) important in explaining variation in agriculture productivity than rainfall (see Schlenker and Lobell

ERL 2010 paper for the benchmark analysis on this point, and Lobell et al 2011 Nature Climate Change). So a null result is hard to interpret: are we averaging over women who are affected and women who aren't? Or over seasons that should matter and seasons that don't?

A more sophisticated analysis would focus on women likely to be employed in agriculture (this is observed in most DHS surveys, or more simply could split sample on urban/rural; controlling for that does not do the trick), would focus on rainfall during preceding growing season months (can use various datasets on cropping calendars, eg from FAO: http://www.fao.org/agriculture/seed/cropcalendar/welcome.do), and would include temperature as well as precipitation during those growing season months. Indeed, "drought" is understood by meteorologists to be a function of both rainfall and temperature.

Looking at heterogeneity by factors that moderate suspected channels would seem a more useful cut on heterogeneity than just looking at results by country, in my view.

Thank you for your comments. We wish to clarify that the primary purpose of this study was to replicate the analysis using the methods of Epstein et al. and Cools et al. with an expanded dataset and accounting for spatial autocorrelation. We sought to show that, using the definitions of drought utilized by those papers, we find no relationship between drought and IPV. Proving that there is absolutely no relationship between any form of drought (such as forms of drought that account for temperature) and IPV is beyond the scope of this paper. We have updated the text to make this scope clearer, and we have discussed some of the suggested changes as future avenues for research.

While we have used the same definition of drought as Epstein and Cools, we have updated the paper to explore heterogeneities based on household wealth, women's employement, household drinking water sources, and whether the household was residing in urban or rural areas. This is to use the framework of previous research but focus specifically on women and households that could be more vulnerable due to livelihood and economic contexts that are more drought-affected.

2. The authors results in Fig S1 without the controls for spatial autocorrelation are I think the closest to the model that Epstein et al ran, but they seem to find very different results in Africa. Can this discrepancy be explained? The authors appear to find that drought is protective in SSA, contrary to Epstein who find that drought increases IPV. If they are going to situate their paper as resolving this debate, it seems important to understand how seemingly similar statistical analyses can yield different results.

We have added more text discussing these findings in relation to those findings by Epstein et al. We now emphasize that simply adding additional data to a model identical to the one run by Epstein et al. yields results with no clear drought-IPV relationship, suggesting that the findings by Epstein et al may have been due to

a spurrious correlation between drought and IPV that was only present in the specific DHS surveys that Epstein chose to use.

Reviewer #3:

The connection between climate and violence (IPV) is an important one to theorize and test, and this is the aim of this paper. The data management and analysis appears competent. My main issue is that the model does not track pathways between drought and violence in any proximal way, despite there being both an important set of literature explaining the connections (not cited here) and available variables to do so within the data set (DHS). Specifically, there is compelling and growing evidence that a main pathway linking drought and IPV is the emotional status of the household, particularly womens failures to meet socially prescribed roles. The intervening ecological factor is water availability/security of the household, which can then be linked to drought. Without this included in the model, the paper is just a test of an association improperly theorized against current literature. Drought isn't an issue if you have access to enough water, and in many of the samples tested there is great underlying variation in household water access within drought regions (e.g., households on municipal supply versus others; those with wells versus those using surface sources etc. This may also have a similar pathway through food insecurity.

Some of this literature:

Choudhary, N., Brewis, A., Wutich, A., & Udas, P. B. (2020). Sub-optimal household water access is associated with greater risk of intimate partner violence against women: evidence from Nepal. Journal of Water and Health, 18(4), 579-594.

Stevenson, E. G., Greene, L. E., Maes, K. C., Ambelu, A., Tesfaye, Y. A., Rheingans, R., & Hadley, C. (2012). Water insecurity in 3 dimensions: An anthropological perspective on water and women's psychosocial distress in Ethiopia. Social science & medicine, 75(2), 392-400.

Collins, S. M., Mbullo Owuor, P., Miller, J. D., Boateng, G. O., Wekesa, P., Onono, M., & Young, S. L. (2019). 'I know how stressful it is to lack water!'Exploring the lived experiences of household water insecurity among pregnant and postpartum women in western Kenya. Global Public Health, 14(5), 649-662.

Mushavi, R. C., Burns, B. F., Kakuhikire, B., Owembabazi, M., Vořechovská, D., McDonough, A. Q., ... & Tsai, A. C. (2020). "When you have no water, it means you have no peace": A mixed-methods, whole-population study of water insecurity and depression in rural Uganda. Social Science & Medicine, 245, 112561.

Wutich, A., & Ragsdale, K. (2008). Water insecurity and emotional distress:

coping with supply, access, and seasonal variability of water in a Bolivian squatter settlement. Social science & medicine, 67(12), 2116-2125.

Wutich, A., Brewis, A., & Tsai, A. (2020). Water and mental health. Wiley Interdisciplinary Reviews: Water, 7(5), e1461.

Hatcher, A. M., Weiser, S. D., Cohen, C. R., Hagey, J., Weke, E., Burger, R., ... & Bukusi, E. A. (2020). Food insecurity and intimate partner violence among HIV-positive individuals in rural Kenya. American journal of preventive medicine.

Diamond-Smith, N., Conroy, A. A., Tsai, A. C., Nekkanti, M., & Weiser, S. D. (2019). Food insecurity and intimate partner violence among married women in Nepal. Journal of global health, 9(1).

Brewis, A., Choudhary, N., & Wutich, A. (2019). Household water insecurity may influence common mental disorders directly and indirectly through multiple pathways: Evidence from Haiti. Social Science & Medicine, 238, 112520.

Thank you for your extensive feedback and the suggestions of literature to include. We now dicuss the literature that you have brought to our attention, and added an analysis exploring the effects of drought on IPV for households with seven different types of drinking water supply.

Reviewer #4:

I appreciate the invitation to review the paper, "Reexamining the Effects of Drought on Intimate-Partner Violence." This study addresses an important issue and is generally well-designed, and the manuscript is very well-written. In my view, with some improvements it has the potential to make significant contributions to the climate-violence and climate-health literatures. My comments and questions are as follows: 1. Does spatial autocorrelation account for non-random exposure or does it account for spatial dependence (e.g., spillovers)? (p 2.)

We have now specifically stated that it accounts for non-random exposure

2. The paper is partly framed as a replication of two other studies, using an expanded dataset and slightly different methodology. However, I was unclear why the authors did not take this opportunity to more significantly improve upon the two studies they engage with. In my view, the following would all strengthen the analysis:

a. Standardize the climate variables to account for typical year-on-year variability. The current use of "percentage of normal" has a number of potential pitfalls, including but not limited to the fact that small absolute changes may lead to excessive percentage changes in places with low average precipitation.
b. Control for temperature in all regressions, as seems to be standard in public health and demographic research on climate impacts.

c. Explicitly control for excessive rainfall. Currently the "reference" group conflates normal and excessively-high rainfall whereas a more meaningful reference would be normal rainfall only.
d. Replace sample fixed effects with province-year or district-year fixed effects (or province/district + time trend). In my view, sample fixed effects are not sufficient given the data will permit the more conservative approaches suggested here.

Our goal was to replicated the analyses in the existing literature, and not to take an entirely novel approach. In our opinion, these approaches you suggest would be beyond the scope of this particular paper. However, in response to this feedback, we now more explicitly state that our goal was to use the drought definition operationalized by Cools et al. and Epstein et al. in order to replicated their approach, and that our results should not be taken as conclusive evidence that there is no form of drought associated with IPV, just that rainfall anomalies over a 12-month period are not associated with most forms of IPV. We also mention potential ways to improve the analysis for future research, based on many of the suggestions you have made.

3. Why do the authors focus on country stratified models rather than other potential moderators (e.g., baseline gender inequality?). I realize some such analyses must be left to future research given space constraints, but some rationale would be helpful.

We have updated the analysis to focus on other types of heterogeneity rather than cross-country comparisons that make more sense as potential proxies for differential vulnerability of households to the impacts of drought.

4. On page 10, the authors note the need to control for spatial autocorrelation. While I agree this is important, I would push them to also think about the substantive processes that underline any such statistical relationships. In other words, we need to think about and ideally model the substantive processes that are being detected through these spatial statistics rather than treat them as statistical nuisances.

Thank you for this feedback. We now discuss additional literature and explore many more causality mechanisms highlighting pathways from drought to IPV, such as households dependency on drought-affected water sources.

5. The undefined lookback period for the "controlling behavior" outcome (discussed on page 10) is important. Since this ends up being a featured finding, I would push the authors to explore the robustness of this finding a bit more (e.g., using alternative exposure periods, placebo tests).

We now examine various subsets of women based on categories of vulnerability to drought, and we find no association between drought and controlling behaviors for any subgroup of women. We address this in our discussion section.

Reviewer #5:

This study adds to previous work on the relationship between drought and IPV by more carefully accounting for spatial autocorrelations than some previous work. The study is an ecological one, with all the attendant limitations of not being able to adjust for many other potentially important factors that may change with drought (incl. changes caused by official responses). The investigation adds a small amount to a specific area in examining ecological factors associated with IPV and as such it may not interest a broad readership. Furthermore, it has less interest to medical audience as drought is non-modifiable - at least in the short or medium term.

I have little to suggest for detailed improvements. The justification for the study is not strong enough in my view for a medical journal.

Some other areas could be improved:

1. An explanation of spatial autocorrelation should be added to the introduction. What assumptions need to be met for control for it in spatial spine models?

Thank you for your feedback, we have added this to the introduction section.

2. Are there any other obvious ecological correlations that could be adjusted, even partially?

The most important correlation is spatial autocorrelation. There are not large temporal patterns in IPV or drought, so we did not adjust for any temporal autocorrelation.

3. What is the effect size of low income for men and low income on IPV outcomes in women?

We ran separate regressions by household income quintile, and found no effect statiscitally significant effect of drought on IPV, even among households in the bottom quintile.

4. Was there a protocol for the analysis? Where the thresholds for drought pre-determined?

We determine our drought thresholds based on the previous thresholds used by Epstein et al. and Cools et al. (See Table 1).

5. How was missing data handled? It is not clear.

We have now made that clearer in the manuscript. Individuals with missing data for any of the variables used in the analysis were excluded from the study.

6. Is it correct to comment on the increased risk of controlling behaviors considering the large number of tests conducted? I'm not sure that Bonferroni corrections are the only measure of whether these are chance associations. My view is that a consistent pattern should be present - which it is not, so

> I think these findings are overemphasised. I can see why the authors may have done this as a null study is quite niche and better suited to a more specialized journal.

We agree, and have added some qualifications on the interpretation of this effect to the discussion, especially in light of no pattern emerging for subsets of the population.
